# Our Advertising Department

**Published:** 1876-03

**Authors:** 


OUR ADVERTISING DEPARTMENT.—The wide awake merchant, anxious to dispose of his goods, always employs printer's ink to catch the purchaser's eye. Our patrons will find in our advertising pages much to attract—things both useful, beautiful and novel. Physicians, druggists, and other readers are invited to give them a careful reading, and when articles advertised in our columns are needed, we request that our advertisers shall have precedence over all others.

The druggists of Atlanta are enterprising, energetic business men, well posted, and fully up to the most accomplished in any city. It is with special pleasure that we refer to the large wholesale house of Hunt, Rankin & Lamar, and to our friends Thos. Pullum & Co., Mote Boyd, and Geo. F. Platt. These gentlemen will do all they say, and our friends can find no better or cleverer men to deal with anywhere. Give them an opportunity to show you how pleasant it is to trade with gentlemen.

And our friends wear hats! How poor is the estimate the public places on the possessor of a shabby, unstylish hat! If our medical friends desire to increase their popularity, and pave the way to fortune by donning a superb hat, let them call upon that prince of hatters and good fellows, J. M. Holbrook.

*The Extract of Malt* will, no doubt, be found a valuable adjunct to the long list of restorative means of building up debilitated patients. It merits a trial in suitable cases.

*The Earth Closet*, as a sanitary and hygienic contrivance, is suited to the wants of most of our population especially. They are decent, clean, and contribute to the health of households using them.

*The Elastic Truss*, manufactured by the Pomeroy Truss Company, is largely used and highly recommended.

*The Agre's Truss* we have noticed before. It is a valuable contrivance; said to be the best.

*Codman & Shurtleef*, makers and importers of surgical instruments, have established a national reputation for honest dealing, and our friends can, with confidence, send orders and be fairly treated, both in quality of goods and prices.

*The Buffalo Lithia Waters* in rheumatic gout are highly recommended, and will doubtless be found very beneficial in a large number of cases of this disease. Physicians can note the fact, and recommend their druggists to become agents for the sale of the waters. The name of the proprietor, Thomas F. Goode, is sufficient guarantee for the truth of all that is stated in his advertisement.

Do you like good pictures? How beautiful the house and office when tastefully adorned by beautiful pictures! *E. & H. T. Anthony & Co.*, and *Alexander & Co.*, in our advertising pages, will tell you how to get them.

*Burrington's Dr. Wadsworth's Uterine Elevator* is an instrument extensively used. It should be kept by every druggist.

The vaccine virus from the animal is certainly the best. The *Pennsylvania Vaccine Agency* furnish the best only.

*H. Planten & Son* are manufacturers of all kinds of medicinal capsules, one of the best means of administering nauseous medicines.

*The Indelible Marking Paper* for marking clothing is a great convenience. Read what B. Alexander & Co. say about it.

*Mineral Water* reduced to mass is a new remedy, commended highly by some of our best medical friends, whom we know to be reliable. It is worthy of an impartial test. Our readers will please recommend its claims to their home druggists.

*W. J. M. Gordon*, the great manufacturing chemist of the Northwest, occupies a large space in our columns. His goods are trustworthy and reliable. Druggists and physicians should make a note of this, and give Gordon the benefit of our recommendation by liberal orders.

